# Assessment of DNA damage in relation to heavy metal induced oxidative stress in females with recurrent pregnancy loss (RPL)

**DOI:** 10.1016/j.sjbs.2021.05.068

**Published:** 2021-06-02

**Authors:** May Alrashed, Hajera Tabassum, Nouf Almuhareb, Nourah Almutlaq, Waad Alamro, Samyah T. Alanazi, Fouza K. Alenazi, Lulwah B. Alahmed, Mubark M. Al Abudahash, Naif D. Alenzi

**Affiliations:** a Department of Clinical Laboratory Sciences, College of Applied Medical Sciences, King Saud University, Riyadh, Saudi Arabia; b Research and Laboratories Sector, National Drug and Cosmetic Control Laboratories (NDCCL), Saudi Food and Drug Authority, Riyadh, Saudi Arabia; c Chair of Medical and Molecular Genetics Research, Department of Clinical Laboratory Sciences, College of Applied Medical Sciences, King Saud University, Riyadh, Saudi Arabia

**Keywords:** DNA damage, Total antioxidant status, ICP-MS, Recurrent pregnancy loss

## Abstract

Pregnancy termination consecutively for three or more times during the first trimester is termed as Recurrent pregnancy loss (RPL). In addition to the abnormal karyotype, heavy metal induced oxidative damage may contribute as prominent etiological factor in pregnancy termination. Oxidative stress is considered crucial in etiology underlying RPL with altered antioxidant status and subsequent DNA damage. The current case controlled study investigated Total antioxidant capacity (TAC), DNA damage (8OHdG) and heavy metals in RPL group (n = 30) and the women with successful pregnancies and no cases of miscarriage as control group (30 women). Heavy metals -Antimony (Sb) and Arsenic (As) were measured by Inductively Coupled Plasma Mass spectrophotometry (ICP-MS). There was significant decrease in levels of TAC in RPL group compared to healthy pregnant women (*P* < 0.05). On contrary, elevated levels of As and Sb were observed in RPL group with subsequent increase in the levels of 8OHdG (*P* < 0.001); indicating extensive DNA damage in these patients. Furthermore, increased levels of As and Sb in RPL group were positively correlated with 8OHdG and negatively with total antioxidant capacity. The outcome of the study provides clear insight of the role of metal induced oxidative stress that plays a vital role in the pathophysiology underlying RPL.

## 1 Introduction

Approximately 1–2% of pregnancies are under threat of recurrent pregnancy loss (RPL). Loss of pregnancies consecutively (three or more times) before 20 weeks gestation in the first trimester with fetal weight >500 g is defined as RPL (Giannubilo et al.,2012). RPL results primarily due to chromosomal abnormalities of parents or fetus. Besides genetic factors, uterine and endocrine abnormalities also contributes to its etiology . Despite intensive and thorough investigations in this field, most of the RPL cases are with unidentified causes. Under normal physiological conditions, oxygen is reduced to water during oxidative metabolism in mitochondria. Fewer percentage of oxygen develops into the molecular species which are highly reactive and toxic called oxidants viz. reactive oxygen/nitrogen species or free radicals (Agarwal et al., 2008). When the levels of these free radicals exceeds the normal physiological limit, the oxidative-antioxidant system gets imbalanced resulting in oxidative stress (OS) that subsequently has deleterious action on the cellular components and function including DNA. By nature, cell harbor antioxidants (enzymatic and non-enzymatic) that scavenge these free radicals and disrupts the OS generated in cells (Stadtman and Levine, 2000, Al-Gubory et al., 2010). One of the best method rationalized to evaluate the OS is measurement of the total antioxidant capacity (TAC). Assessment of TAC provides insight of an individual’s antioxidant status in OS linked diseases including RPL. A further insight into the etiology underlying RPL, DNA damage resulting from OS has emerged as vital process responsible for pregnancy termination. Oxidation of DNA results in formation of products like apurinic DNA, oxidized nitrogenous bases etc. Amongst different products, 8-hydroxydeoxyguanosine (8-OHdG) is gaining significance as a universal marker to detect DNA damage.

Human beings are exposed to heavy metals through air, water, and food sources. Few of the heavy metals are known to cause toxic effects on placental functions (Bloom et al., 2012, Omeljaniuk et al., 2018). Antogonistic effect of heavy metals in RPL have been investigated previously. Yet, role of antimony and arsenic in RPL cases are scarce. Furthermore, very little information on evaluation of 8-hydroxydeoxyguanosine (8-OHdG) as a marker of DNA damage following OS in RPL cases is known. No study correlated the association between TAC, 8-OHdG and heavy metal in patients with recurrent miscarriage. It has been hypothesized that increased oxidative damage to DNA on account of heavy metal intoxication and or OS could be one of the underlying cause for RPL. Based on the above perspectives, the present study focused to investigate TAC, 8-OHdG, and heavy metals in women with RPL.

## 2 Methodology

The present Case Controlled Study comprised of two groups of women in the age group of 26–35 years; Control group consisting of healthy pregnant women, (n = 30), and RPL group (n = 30). Patients were recruited from Section of Obstetrics and Gynecology, King Saud Medical City Hospital with ethical approval from hospital ethical committee. Patient consent was obtained before sample collection.

### 2.1 Inclusion criteria

Women aged 26–35 years with normal pregnancy (in their I trimester) detected by normal ultrasound examinations were recruited as controls. All subjects included were without any record of miscarriage. I trimester pregnant women with consecutive abortions with an average of 4–7 were included as RPL group.

### 2.2 Exclusion criteria

Non-pregnant/ pregnant women with uterine abnormalities, endocrinal irregularities (diabetes mellitus, thyroid dysfunction), hypertension, smoking, working in industrial area with metal exposure were all excluded from the study.

### 2.3 Sample collection

5 ml of blood was collected in sterile metal free vaccutainers after obtaining the patient consent. For preparation of serum , the samples were centrifuged at 3000 rpm for 15 min and shifted at −30 °C until further investigations.

### 2.4 Determination of TAC in serum

The antioxidant status was estimated using Total antioxidant capacity assay kit (MK-187), Sigma. Principally, total antioxidant capacity is measured by monitoring reduction of copper at ~ 570 nm and expressed in mM.

### 2.5 Heavy metal analysis by Inductively Coupled Plasma Mass spectrophotometry (ICP-MS)

Serum levels of As and Sb were determined by Inductively Coupled Plasma Mass spectrophotometer (ICP/MS), Agilent Technologies 7700. Prior to ICP/MS, serum was subjected to centrifugation. Approximately serum (400 µl) was added to 2.5 ml of solvent mix consisting of HNO_3_ and Triton × 100. A standard stock solution (1000 ppb) of the heavy metal was prepared. Working standard in range from 0.05 to 100 ppb was prepared from stock for calibration of standard graphs. The levels of heavy metals analyzed were expressed in ppb.

### 2.6 Assessment of DNA damage

DNA damage was assessed by measuring level of serum 8 OHdG using 8-hydroxy 2 deoxyguanosine (8 OH-dG) *in vitro* ELISA Kit (ab201734). Briefly, the assay involved the addition of 8OHdG standards and samples in coated plates followed with incubation for 60 min after the addition of antibody conjugate. Post incubation, TMB substrate (Tetramethylbenzidine) was added and incubated further 30 min. The absorbance is read at 450 nm in a plate reader by adding stop solution. The concentration of 8OHdG was expressed as ng/ml.

### 2.7 Statistical analysis

Sigma Plot software version 12 was run to draw the statistical output. Serum levels of 8-OHdG, TAC and heavy metals were compared by *t*-test. Pearsons correlation was performed to determine correlation between 8-OHdG, TAC and heavy metals investigated between the two groups..

## 3 Result

**Fig. 1:**
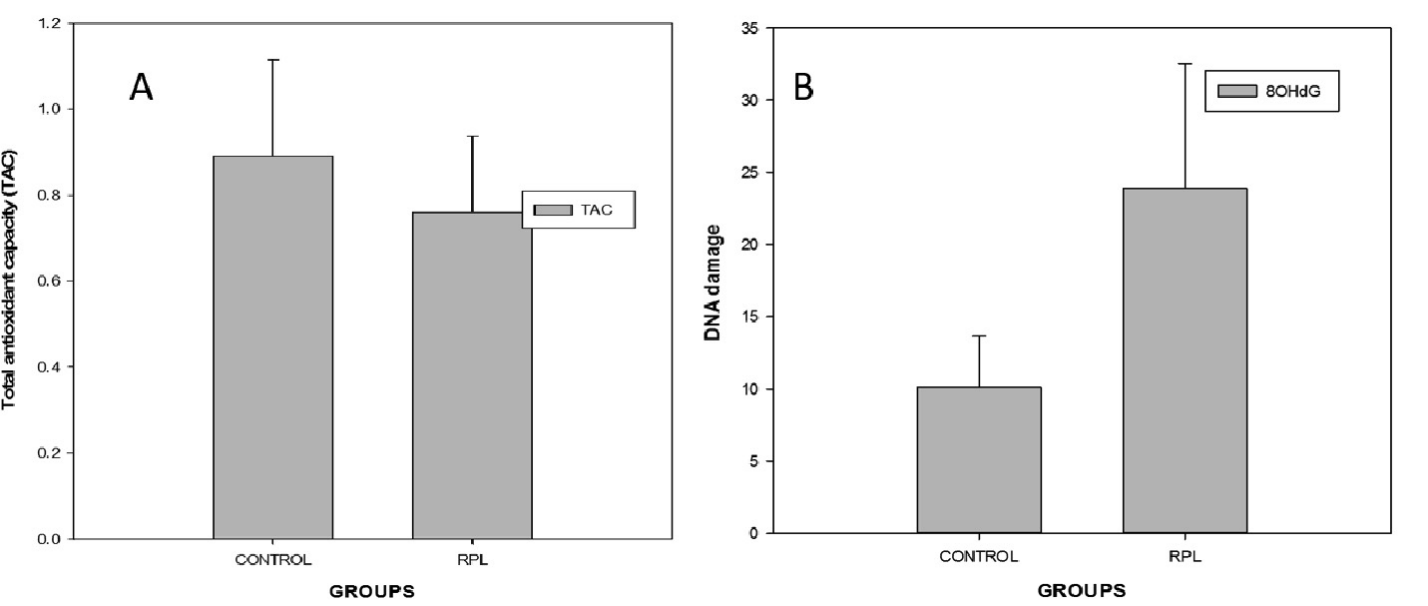
Comparison of TAC and DNA damage in RPL group in relation to Control.

The baseline characteristics of the studied groups are included in Table 1. RPL group exhibited significant increase in levels of FSH, LH and prolactin compared to healthy control. There was no significant change in age, BMI and Hb levels between the two groups studied. Status of TAC, 8-OHdG are shown in Table 2 (Fig. 1). Diminished levels of TAC was found in RPL group than control (P < 0.05). In contrast, serum levels of 8-OHdG decreased in RPL group. The change in level of 8-OHdG was significant at *P* < 0.001.

**Table 1:** Baseline characteristics of the study groups.

	**Control**		**RPL group**	
**Age**	31.20 ± 5.84		29.2 ± 5.3	
**BMI**	29.94 ± 6.05		27.1 ± 3.6	
**Hb%**	11.3 ± 0.81		11.5 ± 0.99	
**Prolactin (ng/ml)**	15.25 ± 1.31		19.65 ± 1.51**	
**FSH (mIU/ml)**	7.35 ± 1.33		11.09 ± 1.02**	
**LH (mIU/ml)**	17.80 ± 0.81		19.30 ± 1.45**	

Values are mean ± SD. **P < 0.001 in comparison with control group.

**Table 2:** Status of TAC and 8-OHdG in Control and RPL groups.

	**Control**	**RPL**
**TAC (mM)**	0.89 ± 0.22	0.76 ± 0.178*
**8OHdG (ng/ml)**	10.12 ± 3.53	23.90 ± 8.64**

Values are mean ± SD ;*P < 0.05 **P < 0.001 in comparison with control group.

**Fig. 2:**
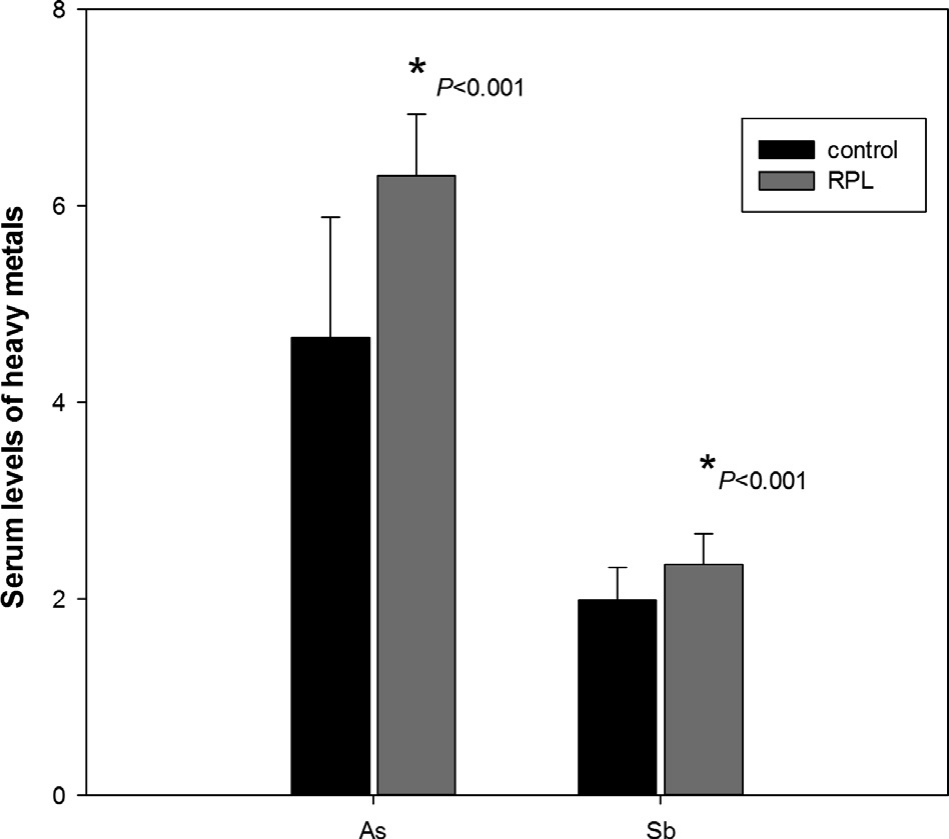
Heavy metals in control and RPL groups.

Serum levels of As and Sb in study groups is illustrated in Fig. 2. The mean serum levels of As (6.31 ± 0.62 ppb) in RPL group was found to be significantly higher than control group (4.66 ± 1.22 ppb) at P < 0.001. Similarly, Sb levels increased significantly in RPL group (2.35 ± 0.31 ppb) compared to control (1.99 ± 0.33 ppb) at *P* < 0.001.

### 3.1 Pearsons correlation of heavy metals with antioxidant status and DNA damage

**Fig. 3:**
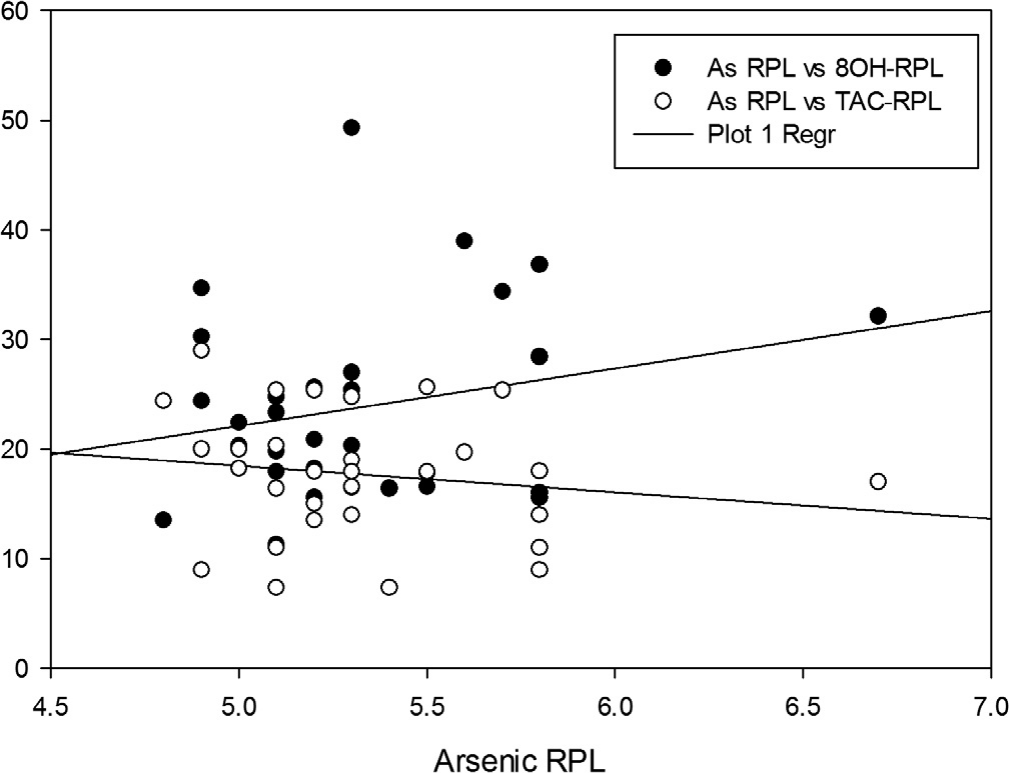
Scatter plot demonstrating negative correlation of Arsenic (As) with TAC and positive correlation with 8OHdG in RPL group.

**Fig. 4:**
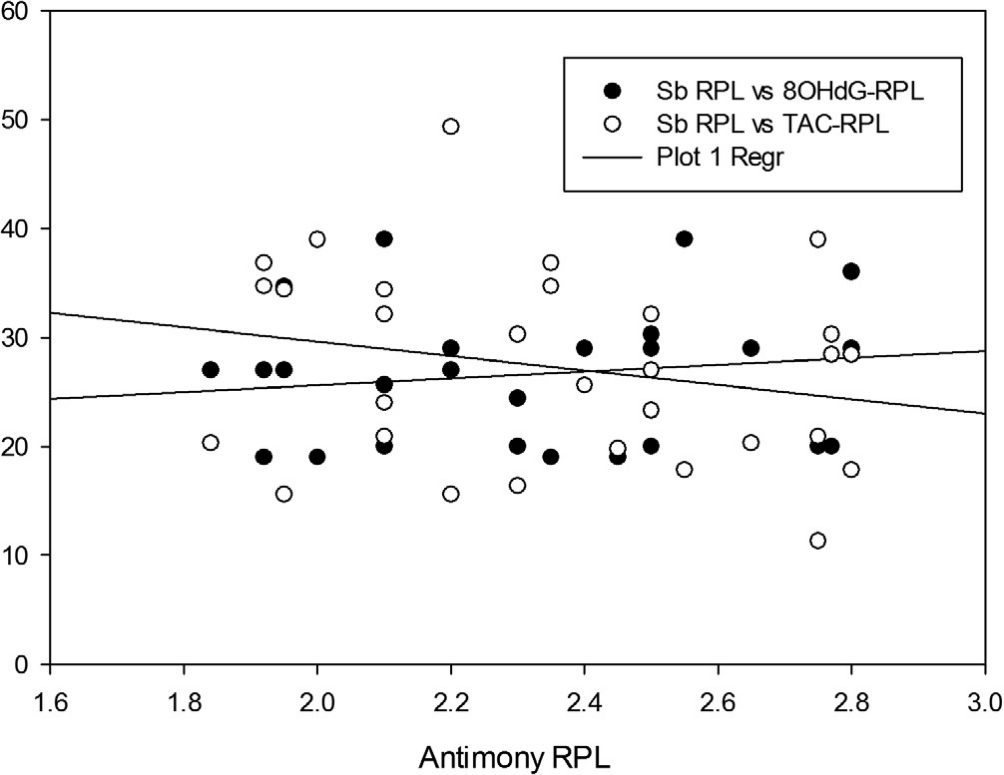
Scatter plot demonstrating negative correlation of Antimony (Sb) with TAC and positive correlation with 8OHdG in RPL group.

Correlation between heavy metals, TAC and 8-OHdG was determined by Pearsons correlation (Table 3**)**. Scatter plots demonstrating negative correlation of Arsenic and Antimony with TAC and 8OHdG in RPL group are illustrated in Fig. 3, Fig. 4**.** Elevated levels of heavy metals and TAC were negatively correlated. As and Sb were positively correlated with DNA damage marker with correlation coefficient values of *r* = 0.32 (*P* < 0.01) and *r =* 0.27(*P* < 0.01) respectively. In contrast, levels of As and Sb exhibited strong negative correlation with TAC; *r* = -0.41 (*P* = 0.001) and *r =* 0.14 (*P* < 0.05) respectively.

**Table 3:** Correlation coefficient value (r) calculated between the heavy metals investigated and oxidative parameters.

	As		Sb	
	Control	RPL	Control	RPL
TAC	*r* = −0.09, *P =* 0.61	***r* = −0.41,** *P =* 0.001*	*r* = −0.008, *P =* 0.64	*r* = 0.14**, *P* < 0.05
8OHdG	*r* = 0.07, *P =* 0.7	*r* = 0.32*, *P* < 0.01	*r* = 0.34, *P =* 0.061	*r* = 0.27*, *P* < 0.01

*P < 0.01,**P < 0.05.

## 4 Discussion

The main paramount findings of the present study was the diminished antioxidant capacity with significant increase in levels of 8OHdG, and heavy metals (As and Sb) in women with recurrent pregnancy loss. Hence, outcome of the current study are in support of hypothesis that oxidative stress generated on heavy metal exposure poses high risk of infertility in women of reproductive age.

Numerous maternal factors (uterine and luteal phase defects, exposure to environmental pollutants/toxicants) and fetal factors (chromosomal abnormalities such autosomal trisomies; the most frequent, monosomy X, and triploidy) predispose women to miscarriage (Simpson, 2007, Franssen et al., 2005). Apart from these factors, oxidative status may also contribute to reproductive anamolies among pregnant females (de Bruin et al., 2002). Increased OS, for instance cause breakage of ds DNA in sperm and/or oocyte leading to recurrent abortions (de Bruin et al., 2002). During normal pregnancy, various antioxidants- enzymatic (SOD, CAT etc) and non-enzymatic normalizes the OS. Hence, disruption of antioxidant control system is suggestive of clinical condition of this disease (Al-Sheikh et al., 2019). Thus, evaluation of TAC provides clear understanding of the role of OS in RM which is evident by the diminished TAC in the current investigation.

Results of this study are indicative of oxidant-antioxidant system shifting in favor of oxidative reactions in RPL women. Supportingly, occurrence of negative correlation between OS and TAC in women with RPL was reported by Yiyenoğlu et al. (2014). Furthermore, the decreased TAC observed here, are in accordance with earlier reports (Al–Sheikh et al. 2019, Ghneim and Alshebly, 2016). Parallely, involvement of OS in RPL cases in relation to control was evidenced by Safronova et al. (2003). Concamitant with ROS, altered and impaired antioxidant status (TAC) was reported by Torkzahrani et al.,2019 with impaired antioxidant defense correlating with spontaneous abortions (Torkzahrani et al., 2019). Parallely, in an Egyptian study, a weekened antioxidant defense with surplus free radicals was reported demonstrating role of oxidative damage in RPL (El-Far et al., 2007).

The main hallmark finding in the present investigation was the significant damage to DNA in RPL group compared to control. Production of 8-OHdG, is amongst the deleterious action of ROS on DNA. Preterm low-birth-weight newborns investigated by Negi et al. (2012), reported an increased levels of 8-OHdG and MDA in these patients (Negi et al., 2012). Additionally, maternal urinary 8-OHdG levels were found higher in early pregnancy with gestational diabetes mellitus (GDM) risk than controls (Peter Stein et al., 2008). Unravelling the association between OS and DNA damage, the current study involved measurement of 8OHdG (DNA damage marker). Enhanced production of 8-OHdG in RPL group is reflective of the DNA damage accountable for pregnancy termination in these patients. The data obtained here, is in accordance with previous finding (Gunduz et al., 2020).

Investigating further into the etiology underlying RPL, - heavy metal were identified as potent toxicant involved in the pregnancy complications including miscarriage. Pregnancy is a state of high sensitivity to toxic substances. A number of sources expell heavy metals into the atmosphere and occupationally or accidentally human are exposure to these metals (Taylor et al., 2016). Women are exposed to heavy metals unknowingly and oftenly every day. Disruption or abnormalities in pregnancy and fetal development are found related to heavy metal toxicity (Bank-Nielsen et al., 2019). Few heavy metals (As, Cd, Pb & Sb) are considered as Endocrine disrupting compounds ‘EDCs’ as they possess endocrine disrupting properties (Bergman et al., 2012). Furthermore, the hazardous effects of these pollutants includes ovulation disorder, failure in implantation leading to pregnancy loss and congenital anomalies (Rebolledo et al., 2011). As and Sb are key toxicants that can concentrate in fetal tissues by passing through the placenta and subsequently to fetal loss. Among metals, As is highly dangerous (Jenardhanan et al.,v2016). The major public health concern is the levels of As permissibility in drinking water. An increased risk of abortions, and neonatal death had been reported with As concentrations (>50 µg/L) in water used for consumption (Rahman et al., 2016). Parallely, a higher rates of spontaneous abortions, stillbirths, and preterm births was reported in population exposed to elevated arsenic concentrations (Ahmad et al., 2001).

Parallel to As, Sb exposure is through air, water, and food sources (Nelson Belzile et al., 2011). Spectacularly, mobile phones are Sb based, which almost all human use including the pregnant women (Huang et al., 2015). Sb exposure could affect liver, lungs, heart and could be neurotoxic too (Cooper and Harrison, 2009, Barbieri et al., 2016). Sb can pass the placenta and could be detrimental to fetal life. (Barbieri et al., 2016). Data on heavy metals investigated in the current report yielded interesting finding. Intriguingly, elevated levels of serum As and Sb in RPL group was observed in relation to control (*P* < 0.001) and in line with earlier finding (Otebhi and Osadolor, 2016). Furthermore, heavy metals investigated demonstrated significant positive correlation with 8-OHdG (DNA damage) and a significant negative correlation with total antioxidant status. The potential toxic metals functions by generating free radicals that eventually precipitate oxidative stress leading to adverse effects on the reproductive physiology among females (Agarwal and Allamaneni, 2004). Based on the outcome of the present study and supportive evidences from the previous published data, heavy metal induced oxidative stress as a major causative factor underlying RPL has been identified.

## 5 Conclusion

Data obtained in the current study is reflective of significant DNA damage owing to generation of OS by heavy metal toxicity among RPL women. Thus, it can be concluded that OS and heavy metals acts as prominent risk factors in etiology of recurrent pregnancy loss. The clear insight into the pathophysiology of RPL would assist gynecologist/obstetrician to develop therapeutic strategies to circumvent the development of these risk factors among pregnant women.

## CRediT authorship contribution statement

**May Alrashed:** Project administration, Supervision, Writing - original draft, Writing - review & editing. **Hajera Tabassum:** Methodology, Writing - original draft, Writing - review & editing. **Nouf Almuhareb:** Methodology. **Nourah Almutlaq:** Methodology. **Waad Alamro:** Methodology. **Samyah T. Alanazi:** Methodology. **Fouza K. Alenazi:** Methodology. **Lulwah B. Alahmed:** Methodology. **Mubark M. Al Abudahash:** Methodology. **Naif D. Alenzi:** Methodology.

## Declaration of Competing Interest

The authors declare that they have no known competing financial interests or personal relationships that could have appeared to influence the work reported in this paper.
